# Phenotypic and molecular characterisations of carbapenem-resistant *Acinetobacter baumannii* strains isolated in Madagascar

**DOI:** 10.1186/s13756-019-0491-9

**Published:** 2019-02-11

**Authors:** Pierrette Landrie Simo Tchuinte, Mamitiana Alain Noah Rabenandrasana, Carole Kowalewicz, Volasoa Herilalaina Andrianoelina, Andriniaina Rakotondrasoa, Zafitsara Zo Andrianirina, Vincent Enouf, Elisoa Hariniaina Ratsima, Frédérique Randrianirina, Jean-Marc Collard

**Affiliations:** 1 Institut Pasteur de Madagascar, Antananarivo, Madagascar; 2 Institut Pasteur, Pasteur International Bioresources network (PIBnet), Plateforme de Microbiologie Mutualisée (P2M), Paris, France; 3 Service de Pédiatrie et Néonatologie, Centre Hospitalier de Soavinandriana, Antananarivo, Madagascar

**Keywords:** *Acinetobacter baumannii*, Carbapenem-resistance, Antimicrobial resistance

## Abstract

**Background:**

The present study aimed to perform a deep phenotypic and genotypic analysis of 15 clinical carbapenem-resistant *Acinetobacter baumannii* (CRAb) strains isolated in Madagascar between 2008 and 2016 from diverse sources.

**Methods:**

CRAb isolates collected from the Clinical Biology Centre of the Institut Pasteur of Madagascar, from the neonatal unit of Antananarivo military hospital, and from intensive care units of Mahajanga Androva and Antananarivo Joseph Ravoahangy Andrianavalona (HJRA) hospitals were subjected to susceptibility testing. Whole-genome sequencing allowed us to assess the presence of antibiotic-resistance determinants, insertion sequences, integrons, genomic islands and potential virulence factors in all strains. The structure of the *carO* porin gene and deduced protein (CarO) were also *assessed in* CRAb isolates.

**Results:**

All isolates were found to be multidrug-resistant strains. Antibiotic-resistance genes against six classes of antimicrobial agents were described. The four carbapenem-resistance genes: *bla*_*OXA-51 like*_, *bla*_*OXA-23*_, *bla*_*OXA-24*_, and *bla*_OXA-58_ genes were detected in 100, 53.3, 13.3, and 6.6% of the isolates, respectively. Additionally, an IS*Aba1* located upstream of *bla*_*OXA-23*_ and *bla*_*ADC-like*_ genes was observed in 53.3 and 66.7% of isolates, respectively. Further, Tn*2006* and Tn*2008* were found associated to the IS*Aba1*-*bla*_*OXA-23*_ structure. An 8051-bp mobilizable plasmid harbouring the *bla*_OXA-24_ gene was isolated in two strains. In addition, 46.7% of isolates were positive for class 1 integrons. Overall, five sequences types (STs), with predominantly ST2, were detected. Several virulence genes were found in the CRAb isolates, among which *two genes, epsA and ptk, responsible for the capsule-positive phenotype, were involved in A. baumannii pathogenesis*.

**Conclusions:**

This study revealed the presence of high-level carbapenem resistance in *A. baumannii* with the first description of OXA-24 and OXA-58 carbapenemases in Madagascar. This highlights the importance of better monitoring and controlling CRAb in Madagascan hospitals to avoid their spread.

**Electronic supplementary material:**

The online version of this article (10.1186/s13756-019-0491-9) contains supplementary material, which is available to authorized users.

## Background

*Acinetobacter baumannii* is a bacterial species that can survive on moist and dry surfaces. *A. baumannii* is widespread in clinical environments, colonizing a variety surfaces (including medical instrumentation), surviving for instance also as a commensal on the skin or hair of hospital staff and patients. The ability of this organism to gain multiple virulence factors and survive in hospital environments for prolonged periods has led to it emerging as a successful worldwide opportunistic nosocomial pathogen. It is responsible for a wide range of infections, such as bacteraemia, sepsis, meningitis and urinary tract infections, and is therefore challenging in terms of infection control by health personnel [1, 2]. This species also has a remarkable propensity for the rapid acquisition of resistance to an extensive range of antimicrobial agents. They can exhibit a major resistance profile, including carbapenems and other *β*-lactam antibiotics, leaving clinicians with limited therapeutic options. Carbapenem-resistant *A. baumannii* (CRAb) have arisen in recent decades. The most common mechanisms for resistance involve enzymatic degradation of the drugs, modification or protection of the target and decreased permeability to or active efflux of antibiotics, often working synergistically. The resistance to carbapenems in *A. baumannii* has been globally related to numerous enzymes, including OXA-type carbapenemases (OXA-51, OXA-23, OXA-24 and OXA-58) and metallo-β-lactamases (IMP and NDM) [3, 4]. Major expression of OXA genes might be facilitated by insertion sequences (ISs), such as IS*Aba1*, IS*Aba4* and IS*Aba125*, which provide an additionally strong promoter [5, 6]. Furthermore, overexpression of the *adeB* gene, tightly regulated by the *adeRS* genes (two-component regulatory system of AdeABC efflux pump family), has also been described implicated in carbapenem resistance in the *Acinetobacter* species [7, 8]. It has also been shown that variation in porins can provide a mechanism to escape from antibacterial pressure. *A. baumannii* intrinsically has a smaller number and size of porins compared with other Gram-negative bacteria (GNB). *The loss of membrane permeability, owing to changes in primary structure, or loss of CarO (carbapenem-associated outer-membrane protein) porin currently are the best characterised causes of intrinsic A. baumannii carbapenem resistance* [9, 10]*. In most of cases, these changes are the result of carO porin gene disruption by various ISs. Based on the variable domains of this porin, this channel is classified into two groups, CarOa and CarOb, where CarOb has been shown to be twice as specific for imipenem than CarOa* [11].

Furthermore, *A. baumannii* exhibits resistance to biocides commonly used in hospitals and laboratories. In addition to the resistance issue, virulence factors have been discovered in *A. baumannii* and a list of virulence factors has been reported by Abbott et al. (2013) [12].

Although a huge number of studies in industrialized countries have reported on the molecular epidemiology and antimicrobial resistance profiles of *A. baumannii*, clinical isolates and, to a lesser extent, virulence factors of individual strains, data on resistance and virulence factors from strains isolated in sub-Saharan countries are scarce. This is especially true for Madagascar where only one study described 53 multidrug-resistant (MDR) OXA-23-producing *A. baumannii* strains collected from various hospitals between 2006 to 2009 [13]. The present study aimed to perform a deep phenotypic and genotypic analysis of 15 clinical CRAb strains isolated in Madagascar between 2008 and 2016. The genetic environment surrounding all carbapenem-resistance determinants, the structure of the gene encoding the CarO porin and the presence of virulence genes have been also investigated.

## Methods

### Bacterial isolates and identification

All CRAb (*n* = 15) isolates collected from clinical samples originating from different studies in Madagascar during the period 2008–2016 (Table 1) and conserved in the biobank of the Institut Pasteur of Madagascar were included in this study. The first identifications were carried out by API 20NE gallery tests and confirmation was conducted by Matrix-Assisted Laser Desorption/Ionization Time-of-Flight Mass Spectrometry (MALDI-TOF MS, Bruker Daltomics, Bremen, Germany).

**Table 1:** Isolate reference number, source, year of isolation and resistance phenotype of carbapenem-resistant *Acinetobacter baumannii* strains isolated in Madagascar between 2008 and 2016

Isolate reference number	Source	Year of isolation	Resistance phenotype Disk diffusion method	E-test (MICs, mg/L)	
				IPM	MEM
AB 346^a^	W	2013	TIC, TIM, PIP, TZP, CAZ, CTX, FEP, GEN, SXT, CIP, MEM, NAL, LVX	> 32	> 32
AB 285^a^	PF	2013	TIC, TIM, PIP, TZP, CAZ, CTX, FEP, GEN, TOB, SXT, CIP, MEM	> 32	> 32
AB 79^a^	W	2016	TIC, TIM, PIP, TZP, CAZ, CTX, FEP, GEN, TOB, AMK, SXT, CIP, MEM, NAL, LVX	> 32	> 32
AB 116^a^	W	2016	TIC, TIM, PIP, TZP, CAZ, CTX, FEP, GEN, TOB, AMK, SXT, CIP, MEM, NAL, LVX	> 32	> 32
AB 184^a^	U	2016	TIC, TIM, PIP, TZP, CAZ, CTX, FEP, GEN, TOB, CIP, MEM, NAL	> 32	> 32
AB 406^a^	BAL	2016	TIC, TIM, PIP, TZP, CAZ, CTX, FEP, GEN, AMK, SXT, CIP, MEM, NAL, LVX	> 32	> 32
AB 334^a^	BAL	2014	TIC, TIM, PIP, TZP, CAZ, CTX, FEP, GEN, TOB, AMK, CIP, MEM, NAL, LVX	> 32	> 32
AB4046^a^	UP	2015	TIC, TIM, PIP, TZP, CAZ, CTX, FEP, GEN, CIP, MEM, NAL	> 32	> 32
AB 141^a^	W	2016	TIC, TIM, PIP, TZP, CAZ, CTX, FEP, GEN, TOB, SXT, CIP, MEM, NAL, LVX	> 32	> 32
AB 006^b^	RS	2015	TIC, TIM, PIP, TZP, CAZ, CTX, FEP, GEN, TOB, CIP, MEM, NAL, LVX	> 32	> 32
AB 153^b^	RS	2015	TIC, TIM, PIP, TZP, CAZ, CTX, FEP, GEN, TOB, CIP, MEM, NAL, LVX	> 32	> 32
AB 142^b^	F	2015	TIC, TIM, PIP, TZP, CAZ, CTX, FEP, GEN, TOB, SXT, CIP, MEM, NAL, LVX	> 32	> 32
AB 187^b^	RS	2015	TIC, TIM, PIP, TZP, CAZ, CTX, FEP, GEN, TOB, CIP, MEM, NAL, LVX	> 32	> 32
AB 1784^c^	BSI	2013	TIC, TIM, PIP, TZP, CAZ, CTX, FEP, GEN, AMK, SXT, CIP, MEM, NAL, LVX	12	> 32
AB 176^d^	SP	2008	TIC, TIM, PIP, TZP, CAZ, CTX, FEP, GEN, AMK, SXT, CIP, MEM, NAL, LVX	> 32	> 32

*MICs* minimum inhibitory concentrations, *TIC* ticarcillin, *TIM* ticarcillin-clavulanic acid, *PIP* piperacillin, *TZP* piperacillin-tazobactam, *CAZ* ceftazidime, *CTX* cefotaxime, *FEP* cefepime, *GEN* gentamicin, *TOB* tobramycin, *AMK* amikacin, *CIP* ciprofloxacin, *MEM* meropenem, *IPM* imipenem, *NAL* nalidixic acid, *LVX* levofloxacin, *W* wound, *PF* pleural fluid, *U* urine, *BAL* bronchoalvealar lavage fluid, *UP* urethral pus, *SP* superficial pus, *RS* rectal swab, *F* faeces, *RS* rectal swab, *BSI* blood stream infection

^a^Strains isolated from biological samples collected at Institut Pasteur of Madagascar

^b^Strains collected from the neonatal unit at Antananarivo military hospital

^c^Strains isolated from the intensive care unit at Mahajanga Androva Hospital

^d^Strains isolated from the intensive care unit at Antananarivo Joseph Ravoahangy Andrianavalona (HJRA) hospital

MICs > 8 mg/L for imipenem and meropenem were considered resistant

### Antimicrobial susceptibility testing

Antimicrobial susceptibilities of the isolates were determined by the disk diffusion method on Mueller-Hinton agar (Bio-Rad, France), as recommended by the Antibiogram Committee of the French Microbiology Society (ACFMS 2015). The following antimicrobial agents were tested: ticarcillin; ticarcillin/clavulanic acid; piperacillin; piperacillin/tazobactam; ceftazidime; cefotaxime; cefepime; meropenem; nalidixic acid; levofloxacin; ciprofloxacin; gentamicin; tobramycin; amikacin; and trimethoprim/sulfamethoxazole. Resistance (Minimum Inhibitory Concentrations, (MICs)) to imipenem and meropenem was determined by Etests (BioMerieux, Marcy l’Etoile, France) according to the manufacturer’s instructions and ACFMS breakpoints (strains displaying MICs > 8 mg/L for imipenem and meropenem were considered resistant).

### DNA extraction and whole-genome sequencing (WGS)

DNA was extracted with the cador Pathogen 96 QIAcube HT Extraction Kit (Qiagen, Paris, France) on a Qiacube HT from 5 mL of liquid cultures grown overnight at 37 °C in Luria-Bertani infusion medium following the manufacturer’s protocol. Purity and DNA quantity were assessed using a Nanodrop spectrophotometer (Thermo Fisher Scientific, Waltham, USA). Illumina sequencing libraries were prepared by with the Nextera XT DNA Sample Kit (Illumina, San Diego, USA) featuring indexed-encoded adapters from Illumina based on the manufacturer’s instructions. The libraries were pooled for sequencing on a NextSeq 500 platform (Illumina) using 2 × 150-bp runs. FqCleaner (version 3.0) was employed to eliminate adaptor sequences (1), reduce redundant or overrepresented reads (2), correct sequencing errors (3), merge overlapping paired reads (4) and discard reads with Phred scores (measure of the quality of identification of nucleobases generated by automated DNA sequencing) < 20. Sequences with < 40 times average coverage after trimming were resequenced to avoid artefacts with respect to allele calling. The Illumina sequence data were assembled utilising Spades software (version 3.9.0).

### Genome annotation and molecular analyses

All assembled genomes obtained were analysed and annotated with online tools and/or open-access databases and manual examinations. A *Rapid Annotations using Subsystems Technology* (RAST) server *was applied for genome* annotation [14]. The antimicrobial resistance gene profile was determined via KmerResistance 2.2 from the Centre for Genomic Epidemiology (CGE) [15]. Mobile elements, including ISs and class 1 integrons, were identified using the software’s ISsaga from ISFinder and Geneious 11.0.5, respectively [16, 17]. The prediction of number and visualization of genomic islands (GIs) were performed through the webserver, IslandViewer4 [18].

#### Distribution of virulence genes

In silico *investigation of certain genes known as virulence factors in A. baumannii (AC nb:* CP018861), *such as cva*C (encoding for colicin V production), *ptk* (encoding a putative protein tyrosine kinase) and *epsA* (encoding a putative polysaccharide export outer-membrane protein) was performed. The genes of *csu* (*csuA, csuB, csuC, csuD and csuE*, encoding the chaperone-usher Csu fimbriae) and *pga loci* (*pgaA, pgaB, pgaC and pgaD*, encoding the polysaccharide poly-N-acetylglucosamine, PNAG), responsible for biofilm formation, were also investigated [19–21].

The complete manual annotation of all resistance and virulence determinants and their genetic environments was performed via Geneious software 11.0.5 (Biomatters, Auckland, New Zealand).

#### CarO amino acid sequence alignment and in silico analyses

*The presence of the car*O porin (AC nb: FJ652396) gene was screened for in all our CRAb isolates. *The nucleotide/predicted amino acid sequences of CarO isoform proteins were determined to type the CarO (CarOa or CarOb) and evaluate gene disruption by ISs. The typing was conducted by sequence alignment of predicted CarO proteins with those already described in the NCBI database (CarOa, AC nb: ZP_05828783 and CarOb, AC nb: ADQ27797) using Geneious software. The amino acid changes observed were analysed with PROVEAN (*http://provean.jcvi.org*/) software, which predicts whether an amino acid substitution, insertion or deletion has any impact on the biological function of a protein.*

### In silico multilocus sequence typing (MLST)

The MLST scheme described by Bartual et al. [22] was performed according to the *Acinetobacter baumannii* MLST (Pasteur) database (https://pubmlst.org/abaumannii/). The assembled sequences of seven housekeeping genes (*cnp62, fusA, gltA, pyrG, recA, rplB* and *rpoB*) were aligned through BLAST (Basic Local Alignment Search Tool), and the aligned sequences were then extracted by comparing them to allele profiles from the *A. baumannii* MLST (Pasteur) database.

### Core genome phylogeny analysis

To investigate genetic relatedness of the CRAb isolates evaluated in this study with those described in the literature, core genome phylogeny was performed with Harvest Suite version v1.1.2 [23]. In addition to the local CRAb panel, a set of five genomes geographically unrelated was included in the analysis. Genomic data utilised in the phylogenetic tree were downloaded from the NCBI (National Centre for Biotechnology Information) database, including the complete genome sequences of the *A. baumannii* isolates ATCC1778 (CP000521), ACICU (CP000863), MDR-ZJ06 (CP001937), A388 (CP024418) and AB0057 (CP001182) [24].

### Nucleotide sequence accession numbers

The 6302-bp, 5551-bp and 6486-bp *A. baumannii* genomic sequences containing Tn*2006*, Tn*2008* and *bla*_OXA-24_ genes and surrounding regions were deposited in the EMBL database under accession numbers LS999837.1, LS999838.1 and LS999872.1, respectively.

## Results

### Antimicrobial susceptibility testing

Table 1 summarizes the source, year of isolation and resistance phenotype of all CRAb isolates studied. The clinical sources from which the strains were isolated were wounds 26.7% (*n* = 4); rectal swabs/faeces 26.7% (*n* = 4); bronchoalveolar lavage fluids 13.3% (*n* = 2) and urine, pleural fluid, urethral pus, superficial pus and blood culture (one sample representing each at 6.7%). All isolates (100%) demonstrated resistance to at least three classes of antibiotics, hence matching with the MDR criteria [21]^.^ The disk diffusion method indicated that all strains (100%) were resistant to ticarcillin, ticarcillin/clavulanic acid, piperacillin, piperacillin/tazobactam, cefotaxime, ceftazidime, cefepime, gentamicin and meropenem. The rate of other resistances was as follows: 93.3% (*n* = 14) for nalidixic acid and ciprofloxacin, 80% (*n* = 12) for levofloxacin, 66.6% (*n* = 10) for tobramycin, 40% (*n* = 6) for amikacin and 60% (*n* = 9) for trimethoprim/sulfamethoxazole. The studied isolates were resistant to imipenem and meropenem with MIC values above 32 mg/L (except AB1784, MIC to IPM = 12 mg/L).

### Detection of drug-resistance genes and related ISs

**Fig. 1:**
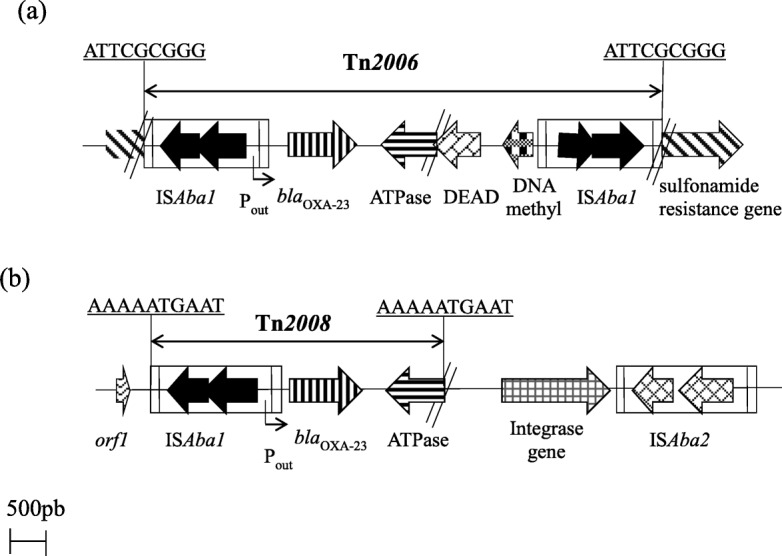
**a** and **b** Schematic representation of two transposons described in this study: (**a**) Tn*2006*; (**b**) Tn*2008* and their genetic environment. The nine-bp target site duplication of Tn*2006* and Tn*2008* are represented underlined. Insertion elements are represented by IS*Aba1* and IS*Aba2*. P_out_: IS*Aba1* promoter; *orf1*: orf encoding for a hypothetical protein; ATPase: gene encoding the putative AAA ATPase; DEAD, gene encoding the putative DEAD helicase

The natural intrinsic *bla*_OXA-51like_ (*bla*_OXA-65_, *bla*_OXA-66_, *bla*_OXA-67_ and *bla*_OXA-69_) gene, as well as three acquired oxacillinases genes, *bla*_OXA-23_, *bla*_OXA-24_ and *bla*_OXA-58_, were present in 100, 53.3% (*n* = 8), 13.3% (*n* = 2) and 6.7% (*n* = 1) of our CRAb isolates, respectively (Table 2). IS*Aba1* was detected upstream all *bla*_OXA-23_ genes, and this was later embedded in Tn*2006* and Tn*2008* transposons, all chromosomally located (Fig. 1).

**Table 2:** Genetic features of the carbapenem-resistant *Acinetobacter baumannii* isolates described in this study

Isolate reference number	ST	Antimicrobial resistance genes	IS*Aba1* + *bla*_OXA-23_	IS*Aba1* + *bla*_ADC_	Number of IS	Transposon distribution
AB 346	2	*bla*_OXA-66_; *bla*_ADC-25_; *aph(3′)-Ia aadA; aac(3)-Ia; aph(6)-Id; aph(3″)-Ib; sul1;sul2, tet(B)*	–	+	17	–
AB 285	1195	*bla*_OXA-67/23/58_; *tet(39) bla*_ADC-106_; *aac(3)-IId*; *sul2; aph(6)Id*	+	–	16	Tn*2008*
AB 79	1196	*bla*_OXA65/24_; *bla*_ADC-52_, *aac(3)-IIa; aph(3″)-Ib; sul2; aph(6)Id*; *bla*_TEM1b_	–	+	13	–
AB 116	79	*bla*_OXA-65/23_; *bla*_TEM-1B_; *aac(3)-IIa;bla*_ADC-52_; *sul2; mphE; aph(3″)-Ib; aph(6)Id; msr(E)*	+	+	20	Tn*2006*
AB 184	1	*bla*_OXA-69_; *bla*_ADC-11_; *ant(3″)-Ia*	–	–	7	–
AB 4066	2	*bla*_OXA66/23_; *bla*_ADC-25_*; tetB; aac(3)-Ia; aph(3′)-Ic; ant(3″)-Ia; sul2; sul1; aph(3″)-Ib; aph(6)Id*	+	+	5	Tn*2006*
AB 334	1196	*bla*_OXA-65/24_; *bla*_TEM-1B_; *aac(3)-IIa;bla*_ADC-25_; *sul2; aph(3″)-Ib;; sul; aph(6)Id*	–	+	7	–
AB4046	2	*bla*_OXA-66_; *bla*_ADC-25_*; tetB; aac(3)-Ia; aph(3′)-Ia; aph(3′)-IC; aph(6)-Id; tetB; ant(3″)-Ia; sul2; sul1; aph(3″)-Ib*	–	+	5	**–**
AB 141	2	*bla*_OXA-66/23_; *bla*_ADC-25_*; aac(3)-Ia; ant(3″)-Ia; aph(3′)-IC; sul2; sul1; tetB; tet(32); aph(3″)-Ib, cmlA1;aph(6)Id*	+	+	5	Tn*2006*
AB 006	1	*bla*_OXA-69/23_; *bla*_ADC-81_; *ant(3″)-Ia*	+	–	2	Tn*2006*
AB 153	1	*bla*_OXA-69/23_; *bla*_ADC-10_; *ant(3″)-Ia*	+	–	4	Tn*2006*
AB 142	2	*bla*_OXA-66/23_; *bla*_ADC-25_; *aac(3)-Ia ant(3″)-Ia; aph(3′)-Ia; aph(3″)-Ib sul1; sul2;tetB;aph(6)-Id; mphE; msr(E)*	+	+	5	Tn*2006*
AB 187	1	*bla*_OXA-69_; *bla*_ADC-81_; *ant(3″)-Ia*	–	–	2	–
AB 1784	2	*bla*_OXA-66_; *bla*_ADC-25_*; tetB; aac(3)-Ia; aph(3′)-IC; ant(3″)-Ia; sul2; sul1; aph(3″)-Ib; aph(6)-Id*	–	+	4	–
AB 176	2	*bla*_*OXA-66/23*_ *; bla*_*ADC-25*_ *; aac(3)-Ia; ant(3″)-Ia; aph(3”)Ia; aph(3″)-Ib; aph(6)Id; sul1; sul2; tetB*	+	+	4	Tn*2006*

+; presence of the indicated structure; −: absence of the indicated structure; ST, sequence type; IS, insertion sequence

**Fig. 2:**
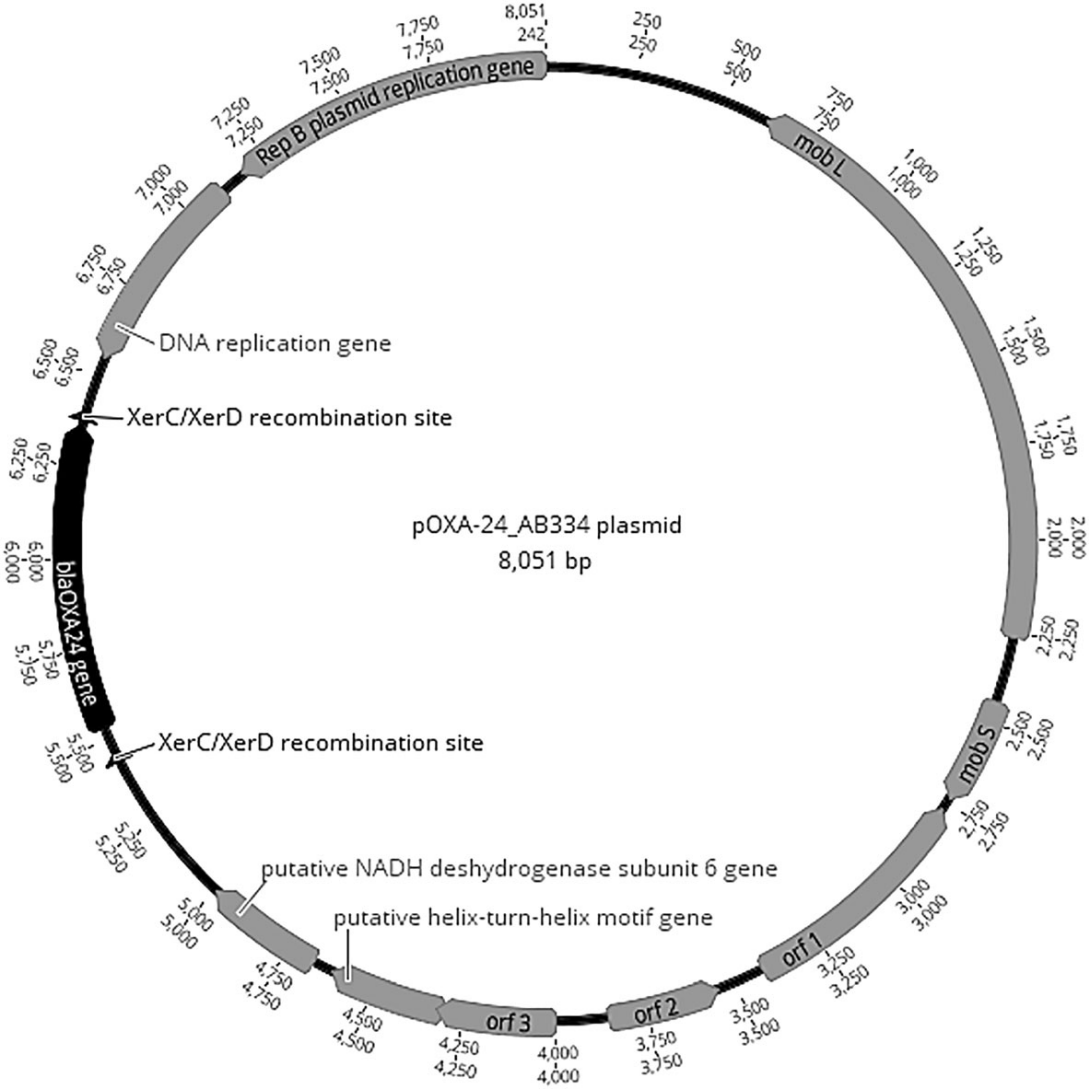
Circular map of the pOXA-24_AB334 plasmid. The two mobilization genes are represented by *mobL* and *mobS*. *orf1*, *orf2* and *orf3*: genes encoding for hypothetical proteins

**Fig. 3:**
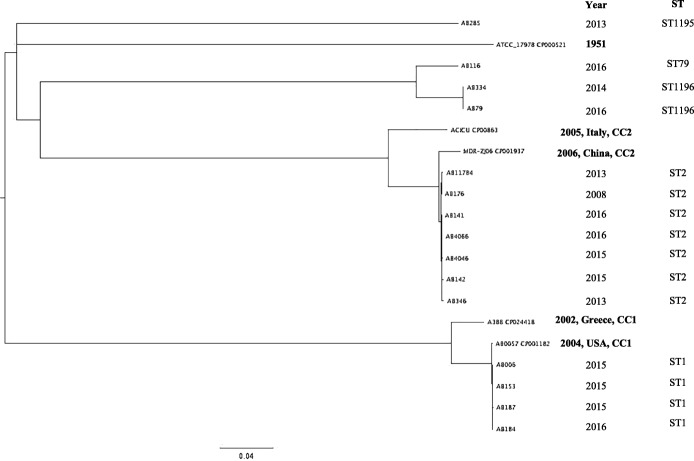
Phylogenetic analysis of 15 *A*. *baumannii* isolates. The phylogenetic tree was constructed with the Harverst suite for core genome alignment and visualization

The two *bla*_OXA-24_ genes flanked by the XerC/XerD recombination sites were located on a small plasmid (8Kb) designated pOXA-24_AB334. This harboured nine open-reading frames (Fig. 3), among which two replicon genes (*repA* and *repB*) and two plasmid mobilization genes (*mobL* and *mobS*) were found. Comparative analysis revealed that pOXA-24_AB334 was 99.1% identical with pABUH3a-8.2 (NZ_AYFH01000048.1) detected in the clinical *A. baumannii* UH 7607 strain isolated in the USA. Further, these two plasmids belong to the small Rep-3 superfamily usually described in *A. baumannii* [25].(Fig. 2).

The KmerResistance 2.2 results revealed the presence of several others antibiotic-resistance genes (Table 2). The *ampC Acinetobacter*-derived cephalosporinase *bla*_ADC_-like gene was established in all CRAb isolates. Sixty-six percent (*n* = 10) of the *bla*_ADC_-positive isolates were associated with IS*Aba1* upstream of the *bla*_ADC_ genes. The variants identified were exhibiting more than 98% identity with *bla*_ADC-10_, *bla*_ADC-11_, *bla*_ADC-25_, *bla*_ADC-52_, *bla*_ADC-81_ and *bla*_ADC-106_ genes. All strains harboured at least one gene involved in aminoglycoside resistance (*aac(3)-Ia; aac(3)-IIa; aph(3’-Ia; aph(3′)-Ic; aph(3″)-Ia; aph(3″)-Ib; aph(6)-Id, ant(3″)-Ia*). Genes encoding resistance to tetracycline and/or minocycline (*tet(B), tet39*), sulphonamides (*sul1, sul2*), macrolides (*msrE, mphE*), β-lactams (*bla*_TEM1b_) and chloramphenicol (*cmlA1*) resistance were also detected.

### Mobile genetic elements

Investigation of mobile genetic elements showed that 46.7% (*n* = 7) of isolates were positive for class 1 integrons (Table 2), which were all embedded in chromosomal genomic resistance island, *Ab*GRI2–1. Gene cassette (GC) arrays encoded for resistance to aminoglycosides (*aac(3)-Ia*, *ant(3″)-Ia*) and for proteins with unknown functions (*orfP1-orfP2-orfQ)*. ISs screening in all genomes analysed demonstrated a large diversity present, mainly of the IS*3*, IS*4*, IS*30,* IS*256* and IS*L3* families.

### Genome annotation and GIs

The genome length for our *A. baumannii* isolates were approximately 4 million bp with an annotation average coverage of 50%, and the number of coding DNA sequences per genome was approximately 3500. The GIs of all genomes were detected by IslandPath-DIMOB software and their number varied from 3 to 8. The visualization of the genetic structures of these GIs revealed the presence of antibiotic resistance genes (*aph(6)-Id, aph(3′)-Ib*, *aac(3)-Ia*, *sul1*, *bla*_TEM_); genes encoding multidrug transporters (*tetA* and *qacE∆1*); mercuric- and arsenic-resistance determinants; transposable elements, including IS (IS*Aba1*, IS*26*, IS*Vsa3* and IS*1006*); and the Tn*7* transposon.

#### Distribution of virulence genes

Additional file 1: Table S1 summarizes the distribution of virulence genes described in all CRAb isolates described herein. The distribution of virulence genes revealed the presence of *cvaC, csuB, csuC, csuD, csuE pgaA, pgaB, pgaC and pgaD genes in all isolates assessed. The csuA was detected in 86% of strains. Interestingly, the two genes, epsA and ptk, responsible for the capsule-positive phenotype involved in the pathogenesis of A. baumannii* [19] *were found in all CRAb strains analysed****.***

### Analysis of the carO porin gene and CarO isoform proteins

*All strains harboured an intact structure of the carO porin gene. The predicted amino acid sequence showed that CarOa and CarOb were distributed among 20% (n = 3) and 53.3% (n = 8) of our CRAb isolates, respectively (*Table 1*, Additional file* *1**). However, a point mutation in the CarOb protein (S214 T) in AB184, AB006, AB 153 and AB 187 isolates was detected. PROVEAN analysis uncovered that this amino acid substitution had a neutral impact in terms of protein function.* No disruption of the carO porin gene by an IS suggests that the CarO porin transporter was not implicated in carbapenem resistance within our isolates.

### Genotype analysis and phylogenetic tree construction

In order to elucidate the different genotypes of CRAb isolates circulating in Madagascar, an MLST scheme based on the Pasteur database was employed. The results revealed that the isolates were clustered into five different genotypes or STs, mostly represented by ST2 (*n* = 7) followed by ST1 (*n* = 4). Three strains had new allelic profiles and novel ST numbers (ST1195 for AB285 and ST1196 for AB79/AB334) assigned (Table 2).

The phylogenetic tree analysis indicated that all strains belonging to the same ST (ST1, ST2 or ST1196) clustered together. The strains belonging to ST1 (*n* = 4) clustered with AB00057 isolated from the USA in 2004. These four strains recovered in 2015 and 2016 all originated from Antananarivo. Strains belonging to ST2 (*n* = 7) isolated from Antananarivo and Majunga between 2008 and 2016 clustered with MDR-ZJ06, widespread throughout China [26]. Antananarivo and Majunga are separated by 565 km and our results suggest a permanent circulation of ST2 genotype in Madagascar. The strains belonging to the new ST (ST1195 and ST1196) were phylogenetically distinct from the others belonging to ST1 and ST2 (Fig. 3).

## Discussion

Carbapenem resistance in *A. baumannii* is based on the production of carbapenemases or synergistic effects between carbapenemases, porin modifications or loss or modification of the penicillin-binding proteins [26, 27]. In this study, 53.3, 13.3 and 6.7% of 15 CRAb isolates harboured the carbapenemases *bla*_OXA-23_, *bla*_OXA-24_ and *bla*_OXA-58_ genes, respectively (Table 2). The carbapenemases (OXA-23, OXA-24 and OXA-58, respectively) encoded by these genes have been described to confer a reduced susceptibility to carbapenems [28, 29]. Like reported elsewhere, the *bla*_*OXA-23*_ gene in our CRAb isolates was upregulated by an IS*Aba1,* which provides a strong promoter [21, 30, 31]. This is the first report of OXA-24 and OXA-58 carbapenemase-producing *A. baumannii* in Madagascar. This latter was co-expressed with OXA-23 in the AB285 isolate.

Additionally, the OXA-51-like gene was determined in all CRAb isolates. The derived oxacillinase (OXA-51-like) confers a high level of resistance to carbapenems only when it is overexpressed [32]. Nevertheless, high-level MICs of imipenem and meropenem was exhibited by some of strains in which the OXA-51 gene was not upregulated (Table 2), and this could be explained by other mechanisms, such as the reduced expression of penicillin-binding protein (PBP2) or the loss of the particular outer-membrane proteins related to carbapenem resistance, like those highlighted by the results of Fernández-Cuenca et al. [26].

The acquisition and dissemination of the resistance genes are largely owing to the actions of mobile genetic elements (MGEs) [33]. This study described MGEs, such as transposable elements (IS*Aba1*, Tn*2006* and Tn*2008*), class 1 integrons, AbaR-type genomic resistance islands (AbGRI2–1) and plasmids [34]. In accordance with several others reports, these genetic structures could disseminate antibiotic resistance determinants between bacteria cell and thus compromise antibiotic treatment, including those with clinical relevance [34–36].

A striking feature of this study is the large number of antibiotic-resistance genes found (Table 2). The resistance to third-generation cephalosporins and quinolones observed was associated with the presence of an *ampC* gene [21] and the S83 L substitution in the *gyrA* gene, respectively.

Typing and phylogenetic analysis have shown that the majority of strains (11/15) assigned to ST1 and ST2 were closest to isolates belonging to globally disseminated clonal complexes I and II (CC1 and CC2), respectively. A previous study conducted in Antananarivo by Andriamanantena et al. *(*2010) demonstrated the circulation of two *A. baumannii* genotypes (A and B typed by rep-PCR) in clinical settings between 2006 and 2009 [13] but the sequence types of these two genotypes were not investigated and could therefore not be correlated with those of our isolates.

The presence of the most important virulence genes (Table 1, Additional file 1) in almost all CRAb isolates is in agreement with their clinical importance and moreover confirmed they are all potential biofilm producers.

## Conclusions

The deep molecular analysis of our clinical CRAb strains allowed for the description of the newly acquired carbapenemases (OXA-24, OXA-58) not previously described in Madagascar and highlighted the potential role of mobile elements, such as transposons and plasmids, in the dissemination of carbapenem resistance. Different studies, including ours, have provided evidence for the circulation of carbapenemase-producing strains (OXA23/24/58; NDM-1/4) in Madagascar [37]. Such circulation, both in Madagascar and the Indian Ocean region, is becoming worrisome and should be better monitored and controlled in order to avoid a rise in the incidence of untreatable nosocomial infections, especially within intensive care units.

## Additional file

Additional file 1: **Table S1.** Distribution of porins, efflux pumps and virulence genes among the fifteen carbapenems-resistant *Acinetobacter baumannii* strains. (DOCX 17 kb)

## Abbreviations

*A. baumannii*: *Acinetobacter baumannii*
ABC: ATP-binding cassette
AC nb: Accession number
ACFMS: Antibiogram Committee of the French Microbiology Society
ADC: *Acinetobacter*-derived cephalosporinase
AMK: Amikacin
BLAST: Basic Local Alignment Search Tool
CarO: *Carbapenem-associated outer-membrane*
CAZ: Ceftazidime
CGE: Centre for genomic epidemiology
CIP: Ciprofloxacin
CRAb: Carbapenem-resistant *Acinetobacter baumannii*
CRISPRs: Clustered regularly interspaced short palindromic repeats
*csu*: Chaperone-usher
CTX: Cefotaxime
*epsA*: Polysaccharide export outer membrane protein
FEP: Cefepime
GCs: Gene cassettes
GEN: Gentamicin
GIs: Genomic islands
GNB: Gram-negative bacteria
HJRA: Joseph Ravoahangy Andrianavalona (HJRA) hospital
IPM: Imipenem
ISs: Insertion sequences
LVX: Levofloxacin
MDR: Multidrug-resistance
MEM: Meropenem
MFS: Major facilitator superfamily
MGE: Mobile genetic element
MICs: Minimum Inhibitory Concentrations
MLST: Multilocus sequence typing
NAL: Nalidixic acid
NCBI: National Centre for Biotechnology Information
PIP: Piperacillin
PNAG: Poly-N-acetylglucosamine
*ptk*: Protein tyrosine kinase
RAST: *Rapid annotations using subsystems technology*
RND: Resistance nodulation division
SMR: Small multidrug resistance
STs: Sequences type
TIC: Ticarcillin
TIM: Ticarcillin-clavulanic acid
TOB: Tobramycin
TZP: Piperacillin-tazobactam
WGS: Whole genome sequencing
