# Supplementary material for: Phenotypic and molecular characterisations of carbapenem-resistant Acinetobacter baumannii strains isolated in Madagascar
Source: Antimicrob Resist Infect Control. 2019 Feb 11;8:31. doi: 10.1186/s13756-019-0491-9 (PMC6371490; doi:10.1186/s13756-019-0491-9)
Supplement: Supplementary file 1 — Table S1. Distribution of porins, efflux pumps and virulence genes among the fifteen carbapenems-resistant Acinetobacter baumannii strains. (DOCX 17 kb) [file 13756_2019_491_MOESM1_ESM.docx]

**Additional file 1: Table S1.** Distribution of porins, efflux pumps and virulence genes among the fifteen carbapenems-resistant *Acinetobacter baumannii* strains

| Isolate **reference number** | **Porine** | **Virulence genes** | | | | | | | | | | |
| --- | --- | --- | --- | --- | --- | --- | --- | --- | --- | --- | --- | --- |
|  | CarO  variant | *cvaA* | *csuA* | *csuB* | *csuC* | *csuD* | *pgaA* | *pgaB* | *pgaC* | *pgaD* | *epsA* | *ptk* |
| AB 346 | CarOb | **+** | **-** | **+** | **+** | **+** | **+** | **+** | **+** | **+** | **+** | **+** |
| AB 285 | CarOb | **+** | **-** | **+** | **+** | **+** | **+** | **+** | **+** | **+** | **+** | **+** |
| AB 79 | CarOa | **+** | **+** | **+** | **+** | **+** | **+** | **+** | **+** | **+** | **+** | **+** |
| AB 116 | CarOa | **+** | **+** | **+** | **+** | **+** | **+** | **+** | **+** | **+** | **+** | **+** |
| AB 184 | CarOb^1^ | **+** | **+** | **+** | **+** | **+** | **+** | **+** | **+** | **+** | **+** | **+** |
| AB 4066 | CarOb | **+** | **+** | **+** | **+** | **+** | **+** | **+** | **+** | **+** | **+** | **+** |
| AB 334 | CarOa | **+** | **+** | **+** | **+** | **+** | **+** | **+** | **+** | **+** | **+** | **+** |
| AB 4046 | CarOb | **+** | **+** | **+** | **+** | **+** | **+** | **+** | **+** | **+** | **+** | **+** |
| AB 141 | CarOb | **+** | **+** | **+** | **+** | **+** | **+** | **+** | **+** | **+** | **+** | **+** |
| AB 006 | CarOb^1^ | **+** | **+** | **+** | **+** | **+** | **+** | **+** | **+** | **+** | **+** | **+** |
| AB 153 | CarOb^1^ | **+** | **+** | **+** | **+** | **+** | **+** | **+** | **+** | **+** | **+** | **+** |
| AB 142 | CarOb | **+** | **+** | **+** | **+** | **+** | **+** | **+** | **+** | **+** | **+** | **+** |
| AB 187 | CarOb^1^ | **+** | **+** | **+** | **+** | **+** | **+** | **+** | **+** | **+** | **+** | **+** |
| AB 1784 | CarOb | **+** | **+** | **+** | **+** | **+** | **+** | **+** | **+** | **+** | **+** | **+** |
| AB 176 | CarOb | **+** | **+** | **+** | **+** | **+** | **+** | **+** | **+** | **+** | **+** | **+** |

+: presence of indicated gene; -: absence of indicated gene; ^1^, carbapenem outer membrane protein with point mutation S214
